# Men’s Knowledge of Obstetric Danger Signs, Birth Preparedness and Complication Readiness in Rural Tanzania

**DOI:** 10.1371/journal.pone.0125978

**Published:** 2015-05-07

**Authors:** Furaha August, Andrea B. Pembe, Rose Mpembeni, Pia Axemo, Elisabeth Darj

**Affiliations:** 1 Department of Obstetrics and Gynaecology, Muhimbili University of Health and Allied Sciences, Dar es Salaam, Tanzania; 2 Department of Women’s and Children’s Health, International Maternal and Child Health, Uppsala University, Uppsala, Sweden; 3 Department of Epidemiology and Biostatistics, School of Public Health and Social Sciences, Muhimbili University of Health and Allied Sciences, Dar es Salaam, Tanzania; 4 Institute of Public Health and General Practice, Norwegian University of Science and Technology, Trondheim, Norway; Tulane University School of Public Health, UNITED STATES

## Abstract

**Background:**

Men’s involvement in reproductive health is recommended. Their involvement in antenatal care service is identified as important in maternal health. Awareness of obstetric danger signs facilitates men in making a joint decision with their partners regarding accessing antenatal and delivery care. This study aims to assess the level of knowledge of obstetric complications among men in a rural community in Tanzania, and to determine their involvement in birth preparedness and complication readiness.

**Methods:**

A cross-sectional survey was conducted where 756 recent fathers were invited through a two-stage cluster sampling procedure. A structured questionnaire was used to collect socio-demographic characteristics, knowledge of danger signs and steps taken on birth preparedness and complication readiness. Data were analyzed using bivariate and multivariable logistic regression to determine factors associated with being prepared, with statistically significant level at *p*<0.05.

**Results:**

Among the invited men, 95.9% agreed to participate in the community survey. Fifty-three percent could mention at least one danger sign during pregnancy, 43.9% during delivery and 34.6% during the postpartum period. Regarding birth preparedness and complication readiness, 54.3% had bought birth kit, 47.2% saved money, 10.2% identified transport, 0.8% identified skilled attendant. In general, only 12% of men were prepared. Birth preparedness was associated with knowledge of danger signs during pregnancy (AOR = 1.4, 95% CI: 1.8-2.6). It was less likely for men living in the rural area to be prepared (AOR=0.6, 95% CI; 0.5-0.8).

**Conclusion:**

There was a low level of knowledge of obstetric danger signs among men in a rural district in Tanzania. A very small proportion of men had prepared for childbirth and complication readiness. There was no effect of knowledge of danger signs during childbirth and postpartum period on being prepared. Innovative strategies that increase awareness of danger signs as well as birth preparedness and complication readiness among men are required. Strengthening counseling during antenatal care services that involve men together with partners is recommended.

## Introduction

Maternal mortality has decreased worldwide in the last ten years; however, in the Sub-Saharan countries it is still high. The World Health Organization (WHO) estimates that, currently, 287,000 women a year die of preventable complications related to pregnancy and childbirth; the majority of these (99%) occur in developing countries and, out of those, 51% occur in the Sub-Saharan region [1]. The main direct causes of maternal deaths are haemorrhage, hypertensive disorders, infections, prolonged labour and unsafe abortion [2].

In Tanzania, antenatal care (ANC) service is free of charge and 96% of pregnant women attend ANC services at least once during pregnancy, while 43% attend the WHO-recommended four visits according to national estimate [3]. The maternal mortality rate (MMR) is estimated to be 454 per 100,000 live births, amounting to 8,000 maternal deaths per year [3]. To achieve the fifth Millennium Development Goal, aiming at a 75% reduction of maternal mortality, Tanzania should have an MMR of 193 per 100,000 live births or less by 2015. In order to address the MMR challenge, Tanzania, in 2002, adopted the Focused Antenatal Care (FANC)[4] model as one of the strategies to reduce maternal mortality. The aims of FANC include early detection of existing diseases, health promotion, prevention of diseases, and counseling on individual birth preparedness and complication readiness (BP/CR). It is expected that when advice on BP/CR is followed, delays in seeking and reaching care will be reduced [5]. The concept of BP/CR is summarized as follows: to in advance identify a skilled birth attendant, plan transportation in advance, save money, identify where to go in case of emergency, and to identify a blood donor[6].

In Southwest Ethiopia, a prospective study was performed to determine the effectiveness of a birth preparedness program and the use of skilled care. This study found that BP/CR was associated with an increased use of skilled birth attendants [7]. A randomized control trial done in northern Tanzania that involved development of birth plans by health care workers and pregnant mothers, showed skilled birth attendance increased compared to the control area [8]. Moreover, a recent systematic review and meta-analysis of 14 studies from developing countries evaluated the impact of a BP/CR intervention involving women’s groups, families and communities. This meta-analysis showed a significant reduction in neonatal mortality but a non-significant reduction of maternal mortality [9].

Studies from East Africa show that women have inadequate knowledge of obstetric danger signs as well as a lack of birth preparedness plans [10–13]. However, involvement of men in reproductive and maternal health education may contribute to a reduction of preventable fatalities as demonstrated in previous studies in Tanzania and Nepal. When men were engaged in different reproductive health programs, it resulted in positive outcomes such as increased condom use, more couples adhering to the program of prevention of mother-to-child HIV transmission and the use of skilled birth attendants [14–18].

As men in many settings, such as in East Africa, hold the financial power in the family and can influence the decision on where women deliver or what to do in case of an emergency, women depend on financial assistance from men for their reproductive and maternal health needs [19–22]. Ugandan women were more likely to have a birth plan if they were escorted by their partner to the emergency obstetric care, and also if their spouses helped with household chores and childcare [23].

Studies on men’s knowledge of danger signs and steps taken for BP/CR are scarce. In Malawi [24], a study on male perception of birth preparedness and severity of danger signs showed that men would promptly seek care in a health facility if a woman had severe bleeding, convulsions or swelling of hands and feet. Still, a quarter of men would go to traditional healers for care of pregnant women in the case of convulsions. This finding indicates that more efforts are needed to make men understand the severity of pregnancy complications. Therefore, for BP/CR to be effective, men, as well as the whole community, must be empowered with the knowledge of danger signs, in order take appropriate action when labour starts and/or if an emergency occurs.

We have not found a study in Tanzania that assessed knowledge of obstetric danger signs and birth preparedness among men. This study aims to assess men’s knowledge and their involvement in BP/CR in the Rufiji district of Tanzania.

## Materials and Methods

### Study Setting

The study was conducted in the Rufiji district in the Pwani region, about 180 km south of Dar es Salaam, the largest city of Tanzania, during the months of July and August 2012. The Rufiji district has an estimated population of 217,000 with the male population being 105,000 and the female, 112,000 as per national census of 2012 [25]. The literacy rate among men in 2012 was 83.1% [3]. Occupation in this district involves subsistence farming of cassava, maize and vegetables as well as small scale commercialized cashew nut farming. There are currently many private motorcycles in the district. These are sometimes used to transport pregnant women to a hospital after negotiating a fee with the motorcycle owner.

The district is made up of 128 villages. Each village has two to four hamlets under the leadership of a hamlet chairperson. There are two hospitals in the district; one public, and one private run by Pentecostal missionaries; where reproductive health services, including ANC, delivery and emergency comprehensive care are provided. In addition, there are four health centers and 52 dispensaries in the district, where pregnant women receive antenatal and delivery care services.

### Study design and Sampling

The study was designed as a cross-sectional descriptive community survey. A two-stage sampling procedure was employed to select the participants of the study. In the first stage, all health facilities in the district were listed. Fourteen health facilities were randomly selected by using a ballot technique. Then, two villages within the catchment population in each health facility were randomly selected. From these villages, all men who had wives/partners who had delivered within the last two years were included in the study. In total, 756 men were approached to take part in an interview.

### Data collection

Data were collected using a pre-tested structured questionnaire developed by Johns Hopkins Program for International Education in Gynecology and Obstetrics (JHPIEGO) and adopted for the Tanzania context[6] Seven medical students with experience of being research assistants and interviewing in previous studies were trained by the first author on how to collect data. The questionnaire included questions related to socio-demographic characteristics such as age, level of education, marital status, and reproductive characteristics, whether he had escorted the partner to a health facility for ANC in the previous pregnancy, residency and if his partner had experienced any obstetric complication in the past two years. Residency is here defined, as rural settlements for those comprised of hamlets and where people engage in agriculture and fishing as the main economic activity. Urban settlements are defined as minor towns, where the majority of people take part in non-agricultural economic activity[26]. In this study we use the expression ‘semi-urban area’ to mean a minor town having both agricultural and non-agricultural activities. Furthermore, questions on knowledge of danger signs during pregnancy, childbirth and the postpartum period were included. The participant was asked to spontaneously mention any dangers signs during pregnancy, delivery or in the postpartum period. Possible options of danger signs during pregnancy included: excessive vaginal bleeding, swollen hands and feet, blurred vision, fits, fever and severe abdominal pain during pregnancy. In the childbirth period, the obstetric danger signs included: excessive vaginal bleeding, severe headache, fits, severe abdominal pain, labour lasting more that 12 hours and retained placenta. For the postpartum period, danger signs were: excessive vaginal bleeding, severe headache, and fits after delivery, foul smelling discharge and fever. Knowledge of at least one obstetric danger sign during each of the period was coded as Yes or No.

For birth preparedness practices in a previous pregnancy, the man was considered well prepared for BP/CR when at least three of the following six practices had been followed: had identified birth kit, had prepared birth kit, had identified a skilled attendant, had saved money, knew where to go in case of an emergency, had prepared transportation in advance, or had contacted a blood donor in advance. If less than three of these practices had been followed, then he was considered less prepared. The three out six was chosen because previous studies have used the same procedure [13]. Additionally, most people usually buy materials such as gloves, surgical blades and syringes, as part of birth preparedness. In this study we call this practice ‘identifying birth kit’, though it is not included in the concept of BP/CR. Once data were collected, the first author and the research assistants would, on a daily basis, review each questionnaire to make sure they were properly completed. If there were any missing data, the participants were contacted the next day to complete the missing information.

### Data Analysis

Data collected were coded, entered into a database, and statistical analyses were performed using SPSS Version 18. To identify factors associated with birth preparedness, bivariate logistic regression was used. These results were expressed as the Odds Ratio (OR) and with 95% Confidence Interval (CI). Factors that were found to have a *p* value of less than 0.2 in the bivariate analysis were then entered into multivariable logistic regression analysis.

### Ethics Statement

The Muhimbili University of Health and Allied Sciences (MUHAS) Institutional Review Board, known as Senate Publication and Research Committee reviewed the study and granted ethical approval (Ref. No. MU/RP/AEC/Vol XIII). Permission to conduct the study was granted by local leaders at municipal and village level. After the participants who are adults were presented with the objectives and rationale for the study, they were informed of their right to stop the interview at any time if they wished, without giving any reason. They were also informed that their names will not be written on the questionnaires and that the questionnaires would be kept in a safe place to ensure confidentiality of the information provided. Before taking part in the interview, consenting participants signed or put a thumbprint on a written informed consent statement.

## Results

### Socio-demographic of men and spouse’s obstetric characteristics

Out of the 756 men that were invited, 13 men did not want to participate and 18 were followed up three times at their houses but were unavailable. Seven-hundred-and-twenty-five men agreed to be interviewed, giving a response rate of 95.9%. Ninety-three percent of the men interviewed were married with a mean age of 30 (SD 6.6). About two-thirds of the respondents had completed primary education. Participants from the rural area made up the majority of the respondents at 74%. Slightly more than half of the men (51%) had escorted their wives to ANC; seventy-seven percent of the participants’ spouses had delivered in a health facility. Almost 15% of the spouses had experienced an obstetric complication in their last pregnancy (Table 1).

**Table 1:** Socio-demographic characteristics of men and spouse’s obstetric characteristics in the Rufiji District (N = 725).

Characteristic	n (%)
**Age**	
18–24	96 (13.2)
25–34	319 (44.0)
35–44	187 (25.8)
>44	123 (17.0)
**Marital status**	
Single/divorced/widowed	49 (6.8)
Married	676 (93.2)
**Education**	
No education	150 (20.7)
Primary Incomplete	64 (8.8)
Primary Complete	451 (62.2)
Secondary+	60 (8.3)
**Residence**	
Semi urban	188 (25.9)
Rural	537 (74.1)
**Escorted wife to ANC previous pregnancy**	
Yes	376 (51.9)
No	349 (48.1)
**Obstetric complication in previous pregnancy**	
Yes	102 (14.1)
No	623 (85.9)
**Place of delivery previous pregnancy**	
Home	166 (22.9)
Health facility	559 (77.1)

### Knowledge of obstetric danger signs and steps taken in birth preparedness and complication readiness

Fifty-three percent of the men could mention at least one danger sign during pregnancy, 43.9% during delivery and 34.6% during the postpartum period. On knowledge of danger signs, excessive vaginal bleeding during pregnancy was mentioned by 79 men (10.1%); during childbirth, 165 men (22.7%) and post-delivery, 129 men (17.8%). Slightly less than a quarter (21%) mentioned fever during pregnancy but much fewer mentioned this sign for during (5.8%) and after labour (7.2%). Less than five percent mentioned prolonged labour as a danger sign during childbirth (Table 2). There were also other signs mentioned such as anaemia, dizziness, malaria that accounted for 19.7% during pregnancy, 16% during delivery and 10.2% during postpartum period (figures not shown in the table). Regarding BP/CR (Table 3), the most common preparation was to purchase the birth kit (54.3%), then saving money, followed by identification of means of transportation. Few mentioned identification of which health facility to visit in case of emergency as well as identification of a skilled birth attendant. Two hundred and five men, 28.3%, had not done any preparations before childbirth, 43.7% had made one of the supposed birth preparing steps, 16% made two, 11.2% made three, 0.7% made four and only one man (0.7%) made all the preparations. Eighty-seven men 12% had made at least three preparations according to the concept of BP/CR.

**Table 2:** Men’s knowledge of danger signs during pregnancy, childbirth and postpartum.

Obstetric danger sign	n (%)
**During Pregnancy**	
High fever	157 (21.6)
Severe abdominal pain	107 (14.7)
Excessive vaginal bleeding	79 (10.1)
Fits during pregnancy	70 (9.6)
Severe headache	61 (8.4)
Swollen hands/face	22 (3.0)
Loss of consciousness	15 (2.1)
Blurred vision	6 (0.8)
Knowledge of at least one signKnowledge of at least one sign	389 (53.7)
**During Childbirth**	
Excessive vaginal bleeding	165 (22.7)
Fits	101(13.9)
Retained placenta	65 (9.0)
High fever	42 (5.8)
Prolonged labour	35 (4.8)
Severe headache	29 (4.0)
Loss of consciousness	13 (1.8)
Knowledge of at least one sign	318 (43.9)
**During postpartum**	
Excessive vaginal bleeding	129 (17.8)
High fever	52 (7.2)
Fits	37 (5.1)
Loss of consciousness	10 (1.4)
Foul smelling discharge	8 (1.1)
Severe headache	6 (0.8)
Knowledge at least one sign	251 (34.6)

Multiple responses possible.

**Table 3:** Birth preparedness and complication readiness among men in previous pregnancy.

BP/CR	n (%)
Identified birth kit	394 (54.3)
Saved money	342 (47.2)
Identified transport	74 (10.2)
Identified where to go for emergency	13 (1.8)
Identified skilled attendant	6 (0.8)
Identified blood donor	1(0.1)
Made at least 3 steps	87 (12)

Multiple responses possible.

### Factors associated with birth preparedness and complication readiness

Residency, escorting wife to ANC clinic and knowledge of danger signs during pregnancy was associated with BP/CR in the binary logistic regression analysis. However, there was no association between BP/CR and age, education, marital status. Further, there was an association between knowledge of danger signs during pregnancy and birth preparedness, but no association between birth preparedness and knowledge of danger signs during childbirth and postpartum. After controlling for confounders through multivariable logistic regression, Men who knew at least one danger sign during pregnancy were more likely to be prepared (AOR = 1.6, 95% CI: 1.8, 2.6). In addition, living in a rural area was a negative predictor for being well prepared (AOR = 0.6, 95% CI: 0.5, 0.8) (Table 4).

**Table 4:** Association between selected socio-demographic characteristics, obstetric characteristics, knowledge of danger signs and birth preparedness and complication readiness.

Characteristic	Prepared n (%)	Less prepared n (%)	Bivariate Analysis Unadjusted OR OR (95%CI)	Multivariate Analysis Adjusted OR OR (95%CI)
**Age**				
18–24	10 (10.4)	86 (89.6)	1	
25–34	37 (11.6)	282 (88.4)	0.7 (0.3–1.8)	
35–44	24 (12.8)	163 (87.2)	0.9 (0.5–1.6)	
>44	16 (13.0)	107 (87.0)	0.9 (0.5–1.9)	
**Education**				
No education	20 (13.3)	130 (86.7)	1	
Primary Incomplete	7 (10.9)	57 (89.1)	0.7 (0.3–2.0)	
Primary complete	55 (12.2)	396 (87.8))	0.9 (0.5–1.6)	
Secondary+	5 (8.3)	55 (91.7)	0.5 (0.2–1.7)	
**Marital status**				
Married	83 (12.3)	593 (87.7)	1	
Single/divorced/widowed	4 (8.2)	45 (91.8)	0.4 (0.5–4.5)	
**Residence**				
Semi urban	175 (6.9)	175 (93.1)	1	1
Rural	463 (86.2)	74 (13.8)	**0.5 (0.3–0.8)**	**0.6 (0.5–0.8)** *
**Escorted Wife to ANC in the previous pregnancy**				
No	42 (12.0)	307 (88.0)	1	
Yes	45 (12.0)	331 (88.0)	1.0 (0.6–1.7)	
**Knowledge of at least one danger sign during pregnancy**				
No	47 (14.4)	289 (85.6)	1	1
Yes	40 (10.3)	349 (89.7)	1.5(1.3–2.4)	**1.4(1.8–2.6)** *
**Knowledge of at least one danger sign during delivery**				
No	56 (13.7)	353 (86.3)	1	
Yes	31 (9.8)	286 (90.2)	0.6 (0.3–1.0)	
**Knowledge of at least one danger sign postpartum**				
No	53 (11.2)	421 (88.8)	1	
Yes	34 (13.5)	217 (86.5)	0.3(0.7–1.9)	

*p value<0.05.

**p value<0.001.

## Discussion

Men in this community have a low level of knowledge of obstetric danger signs. Their Birth preparedness and complication readiness was very low. Men were less likely to be well prepared if they resided in the rural area. Further, being prepared was associated with knowledge of danger signs during pregnancy, but no association with the men’s knowledge of danger signs during childbirth and postpartum.

Men who had spouses delivered in the last two years in the community had inadequate knowledge of obstetric danger signs. This finding is in contrast to Kenyan rural men who were found to have a high awareness of obstetric danger signs [27]. The difference may be explained by the fact that in the Kenyan study, men were asked to respond by saying yes or no to 10 obstetric danger signs that were read out to them. As an unexpected finding, there was no association between knowledge of danger signs during delivery and postpartum with being well prepared, but association was observed with knowledge during pregnancy. Previous studies on knowledge of danger signs and BP/CR among women in Uganda showed there was association between danger signs during pregnancy and the postpartum period with birth preparedness. However, there was no association between knowledge of danger signs during childbirth with birth preparedness [13]. Similarly, studies in Guinea and Ethiopia among women have also shown that knowledge on obstetric complication does not translate into being well prepared [13,28,29]. The explanation for this finding could be related to the source of information that they had received regarding the danger signs and birth preparedness. Another explanation may be that men do not participate in ANC regularly, though they are encouraged to accompany their spouses. In addition, studies have also shown that BP/CR interventions on educating women on danger signs alone did not increase institutional delivery[28]. There is therefore a need to rethink further intervention that involve education on knowledge of birth preparedness and complication readiness

In this study, half of the men had escorted their wives to ANC in the last pregnancy. It is not clear whether they also received information that can lead to appropriate action regarding danger signs and birth preparedness. Among the danger signs, we found that knowledge on severe vaginal bleeding was mentioned, especially during childbirth, by nearly a quarter of men (22.7%). This finding is in agreement with another study completed in the same area among women [10] and in Uganda and Ethiopia [13,30]. Moreover, a study from Malawi demonstrated that after being shown ANC cards with illustrations of danger signs, men would seek care if there was a problem of severe bleeding in their spouses [24]. As haemorrhage is a leading direct cause of maternal mortality globally, it could explain the level of knowledge regarding excessive bleeding [2]; additionally, this is readily visible and dramatic sign.

In this community, only 12% of men were prepared for birth and complication readiness. There have been no previous studies completed in Tanzania regarding birth preparedness among men, but in a study done among women in Tanzania, the majority of women were prepared in identifying where to deliver and who would assist the delivery [31]. In our study, more than half of the men mentioned that they bought the materials for childbirth and 47% reported saving money in advance. Tanzanian healthcare policy states that women should deliver free of charge [32]. Even so, the family is usually told to buy these materials, as commonly the health facilities have little or no stock. There is a need to address this issue as a matter of policy so that it becomes clear what the community needs to get ready for delivery and when a complication arises.

Having skilled attendants at birth is very important in reducing maternal mortality [33]. Surprisingly, in our study, 77% of the spouses had delivered in a health facility. This finding is high compared to the average in Tanzania where facility delivery is reported to be at 50% [3]. Similar findings were observed in Ethiopia, Nepal and Uganda where delivery in a health facility was associated with being well prepared or having a birth plan [12,23,34,35]. Furthermore in Malawi, a study has shown that men can be companions during birth and encourage facility delivery if motivated and given appropriate environment[36]. This finding indicates that previous contact with the health system can increase awareness of the importance of facility delivery as shown in Uganda, where men who knew about ANC services, had obtained information from health workers, and their wives had delivery by skilled birth attendants in the last pregnancy, were more likely to accompany their spouses for ANC services [37]. It is therefore important that men are encouraged to attend ANC services and be treated as partners, so together with their spouses they obtain information on BP/CR and hence help in reducing maternal mortality.

This study has shown that living in the rural area makes men less likely to be well prepared for birth preparedness and emergency readiness. Poverty and the long distances to the health facilities may have contributed to this kind of phenomenon. Distance to the health facility has been shown to be a barrier to the use of skilled birth attendants [5,38]. Studies completed in Ethiopia and Tanzania on factors influencing the utilization of maternal care services and place of delivery have shown that living in rural area makes it less likely for women to deliver in a health facility compared to those living in the urban areas [39,40]. There is a need to improve the rural transportation infrastructure and promote economic empowerment in order to facilitate the proper utilization of maternal health services.

### Strengths and Limitations of the study

Regarding limitations, there are several in our study worth mentioning. As this is a cross-sectional study, it is not possible to provide a causal relationship between being well-prepared and other variables. Additionally, there could be a problem of recall bias because the participants were expected to remember events that occurred up to two years before the study took place. We did not obtain information on the parity of their partners and hence the difference in practice of BP/CR depending on parity could not be demonstrated. Another limitation could be related to social desirability especially when men were asked whether they had escorted wife to antenatal clinic. Furthermore, the participants were required to mention, without being shown pictorials, various critical obstetric situations. Providing the participants with images could have given a different result. These limitations mean that caution is required when generalizing the results to other settings. Despite these limitations, our study demonstrates many strengths. One of the strengths of this study was the large number of men who were willing to participate in the study. In addition, we can be confident that our sampling method of randomly selecting eligible men who met the inclusion criteria has provided a reliable representation of the larger population. Men’s knowledge of danger signs and BP/CR are not sufficiently studied and we have added to the knowledge base by completing this study. It was appropriate to collect the perceptions of this group as they are the important decision-makers in family matters [21]. In studying BP/CR, JHPIEGO[6] proposes assessment at different levels, such as at individual, family (including husband), community, facility and policy level. We have focused this study on the husband/spouse view because there is limited knowledge about this in Tanzania, but further studies are needed to assess BP/CR at these other levels.

## Conclusion

There are low levels of knowledge of danger signs during pregnancy, childbirth and the postpartum period among men in a rural district in Tanzania. A very small proportion of men had prepared for childbirth and complication readiness. In addition there was association between being prepared and knowledge of danger signs during pregnancy, but no association between being prepared and knowledge of danger signs during childbirth and postpartum. There is a need to have innovative strategies that increase awareness of danger signs as well as birth preparedness and complication readiness among men. Strengthening counseling during antenatal care services that involve men together with partners could be one of the strategies. Another strategy could be an intervention that introduces Home Based life saving skills training to families on dangers signs and complication readiness.

## References

[1] WHO U, UNFPA, World Bank (2012) Trends in Maternal Mortality 1990–2010. WHO, Geneva.

[2] Say L, Chou D, Gemmill A, Tuncalp O, Moller AB, Daniels J (2014) Global causes of maternal death: a WHO systematic analysis. Lancet Glob Health 2: e323–333. 10.1016/S2214-109X(14)70227-X 25103301

[3] National Bureau of Statistics (NBS) [Tanzania], Macro I (2011) Tanzania Demographic and Health Survey 2010. Dar es Salaam, Tanzania.

[4] MOHSW (2002) Focused Antenatal care, Malaria and Syphilis in Pregnancy, Orientation Package for Service Providers Reproductive and Child Health Section, Dar es Salaam, Tanzania 2002.

[5] Thaddeus S, Maine D (1994) Too far to walk: Maternal mortality in context. Social Science & Medicine 38: 1091–1110. 8042057 10.1016/0277-9536(94)90226-7

[6] JHPIEGO (2004) Monitoring Birth Preparedness and Complication Readiness: Tools and Indicators for Maternal and Newborn Health. Baltimore, MD: JHPIEGO;. Available: http://www.jhpiego.org/files/BPCRtoolkit.pdf. Accessed 2014 Nov 19.

[7] Tura G, Afework MF, Yalew AW (2014) The effect of birth preparedness and complication readiness on skilled care use: a prospective follow-up study in Southwest Ethiopia. Reprod Health 11: 60 10.1186/1742-4755-11-60 25091203 PMC4127036

[8] Magoma M, Requejo J, Campbell O, Cousens S, Merialdi M, Fillipi V (2013) The effectiveness of birth plans in increasing use of skilled care at delivery and postnatal care in rural Tanzania: a cluster randomised trial. Trop Med Int Health 18: 435–443. 10.1111/tmi.12069 23383733

[9] Soubeiga D, Gauvin L, Hatem M, Johri M (2014) Birth Preparedness and Complication Readiness (BPCR) interventions to reduce maternal and neonatal mortality in developing countries: systematic review and meta-analysis. BMC Pregnancy and Childbirth 14: 129 10.1186/1471-2393-14-129 24708719 PMC4234142

[10] Pembe A, Urassa D, Carlstedt A, Lindmark G, Nystrom L, Darj E (2009) Rural Tanzanian women's awareness of danger signs of obstetric complications. BMC Pregnancy and childbirth 9: 12 10.1186/1471-2393-9-12 19323836 PMC2667432

[11] Mutiso S, Qureshi Z, Kinuthia J (2008) Birth preparedness among antenatal clients. East Afr Med J 85: 275–283. 18817024 10.4314/eamj.v85i6.9625

[12] Hailu M, Gebremariam A, Alemseged F, Deribe K (2011) Birth preparedness and complication readiness among pregnant women in Southern Ethiopia. PLoS One 6: e21432 10.1371/journal.pone.0021432 21731747 PMC3120869

[13] Kabakyenga J, Ostergren P-O, Turyakira E, Pettersson K (2011) Knowledge of obstetric danger signs and birth preparedness practices among women in rural Uganda. Reproductive Health 8: 33 10.1186/1742-4755-8-33 22087791 PMC3231972

[14] Msuya SE, Mbizvo EM, Hussain A, Uriyo J, Sam NE, Stray-Pedersen B (2008) Low male partner participation in antenatal HIV counselling and testing in northern Tanzania: implications for preventive programs. AIDS Care 20: 700–709. 10.1080/09540120701687059 18576172

[15] Mushi D, Mpembeni R, Jahn A (2010) Effectiveness of community based Safe Motherhood promoters in improving the utilization of obstetric care The case of Mtwara Rural District in Tanzania. BMC Pregnancy Childbirth 10: 14 10.1186/1471-2393-10-14 20359341 PMC2858713

[16] Mpembeni R, Killewo J, Leshabari M, Massawe S, Jahn A, Mushi D, et al (2007) Use pattern of maternal health services and determinants of skilled care during delivery in Southern Tanzania: implications for achievement of MDG-5 targets. BMC Pregnancy and childbirth 7: 29 18053268 10.1186/1471-2393-7-29 PMC2222241

[17] Mullany BC, Becker S, Hindin M (2007) The impact of including husbands in antenatal health education services on maternal health practices in urban Nepal: results from a randomized controlled trial. Health Education Research 22: 166–176. 16855015 10.1093/her/cyl060

[18] Kululanga L, Sundby J, Malata A, Chirwa E (2011) Striving to promote male involvement in maternal health care in rural and urban settings in Malawi—a qualitative study. Reproductive Health 8: 36 10.1186/1742-4755-8-36 22133209 PMC3245422

[19] Amooti-Kaguna B, Nuwaha F (2000) Factors influencing choice of delivery sites in Rakai district of Uganda. Soc Sci Med 50: 203–213. 10619690 10.1016/s0277-9536(99)00275-0

[20] Kwambai T, Dellicour S, Desai M, Ameh C, Person B, Achieng F, et al (2013) Perspectives of men on antenatal and delivery care service utilisation in rural western Kenya: a qualitative study. BMC Pregnancy and Childbirth 13: 134 10.1186/1471-2393-13-134 23800139 PMC3691751

[21] Pembe AB, Urassa DP, Darj E, Carlsted A, Olsson P (2008) Qualitative study on maternal referrals in rural Tanzania: decision making and acceptance of referral advice. Afr J Reprod Health 12: 120–131. 20695047

[22] Magoma M, Requejo J, Campbell OM, Cousens S, Filippi V (2010) High ANC coverage and low skilled attendance in a rural Tanzanian district: a case for implementing a birth plan intervention. BMC Pregnancy Childbirth 10: 13 10.1186/1471-2393-10-13 20302625 PMC2850322

[23] Kakaire O, Kaye DK, Osinde MO (2011) Male involvement in birth preparedness and complication readiness for emergency obstetric referrals in rural Uganda. Reprod Health 8: 12 10.1186/1742-4755-8-12 21548976 PMC3118172

[24] Aarnio P, Chipeta E, Kulmala T (2013) Men's Perceptions of Delivery Care in Rural Malawi: Exploring Community Level Barriers to Improving Maternal Health. Health Care for Women International 34: 419–439. 10.1080/07399332.2012.755982 23641896

[25] NBS (2013) Population and Housing Census:Dar es Salaam, Tanzania: National Bureau of Statistics (NBS).

[26] NBS (2013) Basic Facts and Figures on Human Settlements, 2012 National Bureau of Statistics (NBS). Tanzania: National Bureau of Statistics.

[27] Dunn A, Haque S, Innes M (2011) Rural Kenyan men's awareness of danger signs of obstetric complications. Pan Afr Med J 10: 39 22187621 PMC3240929

[28] Brazier E, Fiorentino R, Barry S, Kasse Y, Millimono S (2014) Rethinking How to Promote Maternity Care-Seeking: Factors Associated With Institutional Delivery in Guinea. Health Care for Women International 35: 878–895. 10.1080/07399332.2014.916293 24821280 PMC4160277

[29] Hiluf M, Fantahun M (2007) Birth Preparedness and Complication Readiness among women in Adigrat town, north Ethiopia. Ethiopian Journal of Health Development 22: 14–20.

[30] Hailu D, Berhe H (2014) Knowledge about Obstetric Danger Signs and Associated Factors among Mothers in Tsegedie District, Tigray Region, Ethiopia 2013: Community Based Cross-Sectional Study. PLoS ONE 9: e83459 10.1371/journal.pone.0083459 24516516 PMC3916287

[31] Urassa DP, Pembe AB, Mganga F (2012) Birth preparedness and complication readiness among women in Mpwapwa district, Tanzania. Tanzania Journal of Health Research 14: 1–7. 26591746 10.4314/thrb.v14i1.8

[32] MOHSW (2008) The national road map strategic plan to accelerate reduction of maternal, newborn and child deaths in Tanzania 2008–2015. Dar es Salaam: Ministry of Health and Social Welfare.

[33] Campbell OMR, Graham WJ (2006) Strategies for reducing maternal mortality: getting on with what works. The Lancet 368: 1284–1299. 17027735 10.1016/S0140-6736(06)69381-1

[34] Karkee R, Lee AH, Binns CW (2013) Birth preparedness and skilled attendance at birth in Nepal: implications for achieving millennium development goal 5. Midwifery 29: 1206–1210. 10.1016/j.midw.2013.05.002 23751594

[35] Feyissa TR, Genemo GA (2014) Determinants of Institutional Delivery among Childbearing Age Women in Western Ethiopia, 2013: Unmatched Case Control Study. PLoS ONE 9: e97194 10.1371/journal.pone.0097194 24810609 PMC4014613

[36] Kululanga LI, Sundby J, Malata A, Chirwa E (2012) Male involvement in maternity health care in Malawi. Afr J Reprod Health 16: 145–157. 22783678

[37] Tweheyo R, Konde-Lule J, Tumwesigye NM, Sekandi JN (2010) Male partner attendance of skilled antenatal care in peri-urban Gulu district, Northern Uganda. BMC Pregnancy Childbirth 10: 53 10.1186/1471-2393-10-53 20846369 PMC2946269

[38] Gabrysch S, Campbell OM (2009) Still too far to walk: literature review of the determinants of delivery service use. BMC Pregnancy Childbirth 9: 34 10.1186/1471-2393-9-34 19671156 PMC2744662

[39] Mekonnen Y, Mekonnen A (2003) Factors influencing the use of maternal healthcare services in Ethiopia. Journal of Health Population and Nutrition 21: 374–382. 15038593

[40] Mrisho M, Schellenberg JA, Mushi AK, Obrist B, Mshinda H, Tanner M, et al (2007) Factors affecting home delivery in rural Tanzania. Trop Med Int Health 12: 862–872. 17596254 10.1111/j.1365-3156.2007.01855.x

